# Correction Equation for Hemoglobin Values Obtained Using Point of Care Tests—A Step towards Realistic Anemia Burden Estimates

**DOI:** 10.3390/diagnostics12123191

**Published:** 2022-12-16

**Authors:** Gomathi Ramaswamy, Abhishek Jaiswal, Kashish Vohra, Ravneet Kaur, Mohan Bairwa, Archana Singh, Vani Sethi, Kapil Yadav

**Affiliations:** 1 Department of Community Medicine and Family Medicine, All India Institute of Medical Sciences, Bibinagar 508126, India; 2 Employee State Insurance Corporation Medical College and Hospital, Faridabad 121001, India; 3 National Centre of Excellence and Advanced Research on Anemia Control, All India Institute of Medical Sciences, New Delhi 110029, India; 4 Centre for Community Medicine, All India Institute of Medical Sciences, New Delhi 110029, India; 5 Department of Biochemistry, All India Institute of Medical Sciences, New Delhi 110029, India; 6 UNICEF, Regional Office for South Asia (ROSA), Kathmandu 44600, Nepal

**Keywords:** hemoglobin, digital hemoglobinometer, accuracy, correction factor, auto-analyzer, point-of-care tests

## Abstract

Digital hemoglobinometers have been used as point-of-care tests (POCT) to estimate the burden of anemia in community-based studies and national-level surveys in India. As the accuracy of hemoglobin estimated in POCT varies, there is a need for adjustments to the POCT-hemoglobin to ensure they are closer to reality and are comparable. We used data (collected between 2016 and 2020) (N = 1145) from four studies from India: three among pregnant women and 6–59-month-old children from Haryana and the fourth from a national nutritional survey among 1–19-year-old children. We compared the same individuals’ POCT-hemoglobin (capillary blood) and automated hematology analyzers (AHA) hemoglobin (venous blood) and developed a predictive linear regression model to obtain the correction equation for POCT-hemoglobin. We analyzed paired data from 1145 participants. The correction equation for obtaining the true hemoglobin value = 3.35 + 0.71 × POCT-hemoglobin using capillary blood (adjusted R2—64.4% and mean squared error −0.841 g/dL). In comparison with the AHA-hemoglobin, the mean difference of POCT-hemoglobin was 0.2 g/dL, while with the predicted Hb obtained from the correction equation it was 0.01 g/dL. The correction equation was the first attempt at deriving the true hemoglobin values from the POCTs. There is a need for multi-country collaborative studies to improve the correction equation by adjusting for factors affecting hemoglobin estimation.

## 1. Introduction

Worldwide, approximately 1.8 billion individuals are affected with anemia, and the anemia prevalence is disproportionately high in South Asian, West African, and Central African countries [1]. India has more than 50% prevalence of anemia among vulnerable groups such as those under five and school-going children, adolescents, and pregnant and lactating women [2]. According to the World Health Organization (WHO), iron deficiency anemia has been among India’s top ten causes of disability-adjusted life years (DALY) since 2000 [3]. Iron deficiency anemia is the single most important nutritional risk factor leading to 3% of DALY lost in 2013 in India [4]. Acknowledging the high burden of anemia, the Ministry of Health and Family Welfare, Government of India launched the ‘Anemia Mukt Bharat’ program in 2018 with a target of three percent annual reduction in the prevalence of anemia among vulnerable groups [5].

Hemoglobin is a biomarker used to ascertain anemia status based on the cut-offs provided by the WHO [6]. Hemoglobin estimation with an automated hematology analyzer (AHA) using venous blood estimates the hemoglobin level accurately, but it requires a laboratory setting and has feasibility issues, especially for field-based assessments [7]. With advances in healthcare diagnostics, various point-of-care tests (POCT) such as the cyanmethemoglobin method, WHO hemoglobin color scale, red blood cell protoporphyrin method, Sahli’s hemoglobinometer, and digital hemoglobinometers are available for estimation of hemoglobin in field settings [7,8,9,10,11,12,13]. Though the POCTs mentioned above are easy and feasible to use, the analytical validity of these is relatively suboptimal compared to the gold standard AHAs for hemoglobin estimation [8,9,10,11,12,13]. Additionally, the accuracy of POCTs largely depends on the technician’s competence, proper sample collection procedure, and external environmental factors. Studies have reported diverse sensitivity (24% to 90%) and specificity (60–96%) of these POCT devices in the hemoglobin estimation compared to AHA [8,9,10,11,12,13]. In the last two decades, digital hemoglobinometers using capillary blood have been widely used as POCT for estimating hemoglobin, especially in large-scale surveys and primary healthcare settings [2,14,15].

The burden of anemia reported in various national surveys allows for assessing the progress of the anemia control program. Such surveys at different time points help identify progress in various geographics and risk groups. Thus, the information from these surveys enables data-driven policy making for anemia control. In India, the estimates on anemia are available from the National Family Health Survey (NFHS) and the Comprehensive National Nutrition Survey. The NFHS-4 survey was conducted in 2015–2016, the NFHS-5 was conducted in 2019–2021, and the CNNS was conducted in 2016–2018. NFHS-4/5 included a larger sample population and provided national, state, and district-level estimates. CNNS included a relatively small sample and provided national and state estimates only. Though these surveys were conducted in a shorter time interval, there are more than 10-point differences across groups in the prevalence of anemia while comparing NFHS-4/5 and CNNS. NFHS used POCT-digital hemoglobinometer (Hemocue 201) with capillary blood sample for estimation of hemoglobin and CNNS used AHA with venous blood for estimation of hemoglobin. Hence, there are some challenges in comparing the estimates of anemia from different surveys using different techniques for hemoglobin estimation. Considering AHA as an acceptable standard, there is a need to adjust the hemoglobin values estimated in POCT closer to the real values [2,15,16].

One of the approaches to minimize the variation across different hemoglobin estimation methods is by accommodating the correction equation or factor for the hemoglobin values estimated through POCTs to mimic the value that could have been obtained using the gold standard. The data from the existing validation studies comparing POCTs with the gold standard can be used to deduce such correction factors, which can be used elsewhere with caution. The correction factor will be specific for the POCT and population involved in the validation study. Hence, we have attempted to deduce the correction equation for the hemoglobin values estimated in digital hemoglobinometers as a first step toward availing hemoglobin values closer to that obtained from the AHA.

## 2. Materials and Methods

We used the data collected from four studies, comparing the capillary blood hemoglobin estimated in digital hemoglobinometers and venous blood hemoglobin evaluated in an AHA. Three out of four studies were conducted in primary and secondary healthcare facilities in Haryana, India. The fourth study was conducted in the community as part of the CNNS survey in India. CNNS is the first largest population-based nutritional (macro and micro) survey conducted among 0- to 19-year-old children. A subset of study participants from West Bengal enrolled in the CNNS were randomly included in the validation study, which compared venous and capillary hemoglobin. The detailed methodology of these studies is described elsewhere [10,11,12,13]. Pregnant women (2 studies conducted in 2018 and 2019—dataset A and B) [12,13], 6- to 59-month-old children (1 study conducted in 2019–2022—dataset C) [11], and 1- to 19-year-old age group (1 study conducted in 2016–2018—dataset D) [10] were the study participants included in these 4 studies. Uniform exclusion criteria such as the known history of hemoglobinopathies, metabolic disorders, and chronic diseases affecting blood flow were adopted in all four studies.

### 2.1. Gold Standard or Reference Hemoglobin

The gold standard technique for estimating hemoglobin is the direct cyanmethemoglobin technique. However, the requirement of spectrophotometry, environmental issues with cyanide, and the time-consumption process make the direct cyanmethemoglobin method a challenging technique for estimating hemoglobin [7,8]. The AHA is accepted for their accuracy and reliability as they are automated cell counters following the non-cyanide technique. Though AHAs are expensive, they are being used widely in laboratory settings. Most of the published studies which attempted to validate or compare the hemoglobin values estimated in POCTs used hemoglobin estimated in the AHA from venous blood as the reference value [7,8,9]. Hence, we considered the venous hemoglobin values estimated in AHA as the reference standard.

### 2.2. Comparison or Index Test Values

The hemoglobin values estimated in digital hemoglobinometers were considered index values. Digital hemoglobinometers, especially invasive types, are used globally for the estimation of the burden of anemia in population-based surveys and in healthcare settings where AHAs are unavailable. Hence, we have considered the hemoglobin values estimated in digital hemoglobinometers for prognostication of the correction factor. All four studies used similar single-use auto-disabling lancets with 2.2 mm depth and 23 G needle for obtaining capillary blood. Three types of digital hemoglobinometers, Hemocue 201, Hemocue 301, and TrueHb hemometer, were used in the included studies. Here and onward, the term POCT refers to digital hemoglobinometers in this article. The details of POCTs are in Supplementary Figures S1–S3 [17,18,19].

### 2.3. Approaches for Prognosticating the Corrected Hemoglobin Values

#### 2.3.1. Use of Correction Equation

A correction equation derived from predictive regression equations can be used to obtain the true hemoglobin value for the hemoglobin values obtained through POCTs. Such an equation would also be helpful at the individual level to obtain corrected hemoglobin values and make clinical decisions in the field setting where AHA is not available. The correction equation will be specific for the method of hemoglobin estimation and the population involved during the validation study. The following equation based on the stochastic linear regression model can be used to derive the corrected values:

$$y_{i}=\beta_{0}+\beta_{i}x_{i}+\varepsilon_{i}$$

where *β*_0_ is the intercept, *β_i_* is the slope or coefficient, *x_i_* is a predictor of *y_i,_*, and $\varepsilon_{i}$ is the error term. When we apply this to our exercise,

$$True\;Hemoglobin\;value=\beta_{0}\;Constant+\beta_{1}*Hemoglobin\;estimated\;in\;POCT+\varepsilon$$

this equation provides the predicted true hemoglobin values. After deducing the hemoglobin values, the cut-offs have to be applied to determine the prevalence of anemia in the given population.

#### 2.3.2. Use of Validity Measures

The alternate option is to compute the true prevalence of anemia using sensitivity, specificity, and apparent prevalence values obtained from the POCT from any of the published literature. The ideal process would be to assess the accuracy of the POCT compared to the AHA in a subset of the population for each age group, gender, and other physiological conditions such as pregnancy in each survey. This is crucial, especially in large surveys such as demographic health surveys (DHS), where there is a high chance for potential intra- and inter-observer variations. Hence, multisite validation of instruments is recommended in such large surveys. The following Rogan–Gladen estimator can be used to derive the true prevalence.

$$True\;Prevalence=\frac{Apparent\;Prevalence+\left(Specificity-1\right)\;}{Specificity+\left(Sensitivity-1\right)}$$

We attempted to calculate the true prevalence of anemia based on the reports from NFHS-5. The sensitivity and specificity of the POCT (HemoCue 201) used in the NFHS-5 survey are unknown. Hence, for the calculation of true prevalence, we used the sensitivity and specificity values of the Hemocue 201 from the published literature conducted among pregnant women, adult men, and women. However, this is not an ideal approach as it is better to calculate sensitivity and specificity from the subset sample of the large survey. For children under five years of age, only Hemocue 301’s accuracy values are available, and we used the same for true prevalence estimation.

### 2.4. Statistical Analysis

We used Stata 16.0 for the statistical analysis. The hemoglobin values were summarized as mean (SD) after checking for normality. Bland–Altman plot was used to obtain the mean difference and the limits of agreement between AHA-hemoglobin and POCT-hemoglobin. Univariate and multivariate linear regression analysis were performed to derive the regression coefficients for corrected hemoglobin (dependent factor) with a robust command to deal with heteroskedasticity robust standard errors. We adjusted the independent factors such as age, gender (code for male = 1, female = 2), pregnancy (0 = not pregnant, 1 = currently pregnant), and type of POCTs (1 = Hemocue 201, 2 = Hemocue 301, 3 = True Hb hemometer) in the multivariate regression model to access the effect of them on the regression correction equation model. The adjusted R2 of the model is the level of variance, which can be explained by the regression correction equation and used to evaluate the model’s fitness. A residual plot in the form of a scatter plot (ri = yi − ŷi) was used to assess the distribution of predicted values from actual values and the distribution of residuals.

## 3. Results

In total, we included 1145 study participants from four datasets collected at different time points and from different population groups in this exercise. Of 1145, 424 were pregnant women (212 were tested for two different POCTs and hence counted twice), 120 were under-five children, and 601 were children aged 1 to 19 years old. Three types of POCTs, Hemocue 201 in two studies (datasets A and D), Hemocue 301 in three studies (datasets A, B, and C), and TrueHb hemometer (dataset B) in one study, were used.

Table 1 summarizes the studies included in the calculation of the correction equation. The mean difference in hemoglobin between the AHA and POCTs ranges from −0.3 to 0.5 g/dL. Both the highest (0.53 [95% CI: 0.34–0.73]) and the lowest (0.04 [95% CI: −0.12 to 0.20]) mean differences in hemoglobin values were observed in the facility-based studies conducted among pregnant women. In all the studies, the POCTs had lower mean hemoglobin values compared to AHA, except in a study by Ramaswamy G et al. conducted among 6- to 59-month-old children.

Figure 1 describes the correction equation for each type of POCT across various age groups. We can observe that the hemoglobin values are clustered majorly around the best-fit line. Though there are a few outliers in some of the studies, we have retained outliers in the model to accommodate natural variations in the hemoglobin levels.

Figure 2 shows the error graph of the residual plots, where the residual errors are plotted against the predicted hemoglobin values. We observed some patterns in the residual graphical or significant values in the Breusch–Pagan test. Hence, we used hetrodescadacity random standard errors in the regression.

### 3.1. Method 1—Correction Equation (Table 2 and Table 3)

We have combined all the available data (*n* = 1145) and plotted the residuals, scatter plot of hemoglobin values (Figure 3) estimated in AHA against POCTs, and Bland–Altman plot to assess the mean difference (−0.2 g/dL) and limits of agreement (LOA: −1.9, 2.2).

**Table 2:** Correction equation based on multivariable linear regression for each type of point-of-care test.

Device	Model	Regression Correction Equation y = βo (95% CI of βo) + β1(95% CI) * Hb in POCT +..	Adjusted R2 of the Model	Mean Squared Error
Hemocue 301 * (Dataset A, B, and C)	Model 1: Hemoglobin, Age in years, Sex, and Pregnancy status	y = 1.6 (0.9 to 2.3) + Hb × 0.8 (0.7 to 0.8) + age × 0.01 (−0.01 to 0.01) + sex × 0.1 (−0.3 to 0.3) + pregnancy status × 0.7 (0.4 to 0.9)	0.790	0.663
	Model 2: Hemoglobin, Age in years, and Pregnancy status	y = 1.6 (1.1 to 2.1) + Hb × 0.8 (0.7 to 0.8) + age × 0.01 (−0.01 to 0.02) + pregnancy status × 0.7 (0.5 to 0.9)	0.790	0.661
	Model 3: Hemoglobin and Pregnancy status	y = 1.7 (1.2 to 2.2) + Hb × 0.8 (0.7 to 0.8) + pregnancy status × 0.6 (0.4 to 0.8)	0.789	0.661
	Model 4: Hemoglobin	y = 1.7 (1.2 to 2.2) + Hb × 0.8 (0.8 to 0.9)	0.761	0.746
Hemocue 201 * (Dataset A and D)	Model 5: Hemoglobin, Age in years, Sex, and Pregnancy status	y = 5.5 (4.9 to 6.0) + Hb × 0.5 (0.5 to 0.6) + age × 0.03 (0.01 to 0.04) + sex × −0.1 (−0.2 to 0.1) + pregnancy status × −0.6 (−0.9 to −0.3)	0.541	0.740
	Model 6: Hemoglobin, Age in years, and Pregnancy status	y = 5.3 (4.9 to 5.8) + Hb × 0.5 (0.5 to 0.6) + age × 0.03 (0.01 to 0.04) + pregnancy status × −0.7 (−0.9 to −0.4)	0.540	0.740
	Model 7: Hemoglobin and Pregnancy status	y = 5.3 (4.8 to 5.7) + Hb × 0.5 (0.5 to 0.6)	0.529	0.755
	Model 8: Hemoglobin	y= 5.1 (4.7 to 5.6) + Hb × 0.6 (0.5 to 0.6)	0.528	0.759
True Hb hemometer ** (Dataset B)	Model 9: Hemoglobin and Age in years	y = 1.9 (0.6 to 3.3) + Hb × 0.8 (0.7 to 0.9) + age × 0.02 (−0.03 to 0.1)	0.781	0.572
	Model 10: Hemoglobin using True Hb	y = 2.3 (1.4 to 3.2) + Hb × 0.8 (0.7 to 0.9)	0.780	0.569

* Models with the highest three adjusted R along with the model with only Hb value estimated in POCT as the predictor was mentioned in the Table. ** True Hb hemomoeter—data available only among pregnant women; Code: Sex: 1 = male and 2 = female, Pregnancy status 0 = not pregnant, 1 = currently pregnant; Type of POCT: 1 = Hemocue 201, 2 = Hemocue 301, 3 = True Hb hemometer.

**Table 3:** Deriving a correction equation based on multivariable linear regression based on various combinations of point-of-care tests.

Device	Model	Regression Correction Equation y = βo (95% CI of βo) + β1 * Hb in POCT (95% CI) +..	Adjusted R2 of the Model	Mean Squared Error
Hemocue 201, Hemocue 301, and True Hb (Dataset A, B, C, and D)	Model 11: Hemoglobin, Age in years, Pregnancy status, and type of POCT	y = 4.0 (3.7 to 4.4) + Hb × 0.7 (0.7 to 0.7) + age × −0.01 (−0.02 to −0.01) + pregnancy status × 0.3 (0.2 to 0.5) + Type of POCT × −0.3 (−0.4 to −0.2)	0.668	0.787
	Model 12: Hemoglobin, Pregnancy status, and type of POCT	y = 4.0 (3.6 to 4.4) + Hb × 0.7 (0.7 to 0.7) + pregnancy status × 0.3 (0.1 to 0.4) + Type of POCT × −0.4 (−0.5 to −0.3)	0.664	0.796
	Model 13: Hemoglobin and type of POCT	y = 3.9 (3.6 to 4.3) + Hb × 0.7 (0.7 to 0.7) + Type of POCT × −0.3 (−0.4 to −0.2)	0.659	0.806
	Model 14: Hemoglobin and age	y = 3.7 (3.4 to 4.1) + Hb × 0.7 (0.7 to 0.7) + age × −0.01 (−0.02 to −0.01)	0.657	0.812
	Model 15: Hemoglobin	y = 3.3 (3.0 to 3.7) + Hb × 0.7 (0.7 to 0.7)	0.644	0.841
Hemocue 201, 301 (Dataset A, B, C, and D)	Model 16: Hemoglobin in Hemocue 201 or Hemocue 301	y = 3.5 (3.1 to 3.8) + Hb × 0.7 (0.7 to 0.7)	0.630	0.867
Hemocue 201, True Hb hemometer (Dataset B and D)	Model 17: Hemoglobin in Hemocue 201 or True Hb	y = 4.6 (4.2 to 5.1) + Hb × 0.6 (0.6 to 0.6)	0.565	0.764
Hemocue 301 or True Hb hemometer (Dataset A, B, and C)	Model 18: Hemoglobin in Hemocue 301 or True Hb	y = 1.8 (1.3 to 2.3) + Hb × 0.8 (0.8 to 0.9)	0.769	0.704

* Code: Sex: 1 = male and 2 = female, Pregnancy status 0 = not pregnant, 1 = currently pregnant; Type of POCT: 1 = Hemocue 201, 2 = Hemocue 301, 3 = True Hb.

Lin’s concordance correlation value for the whole data is 0.79. The correction equation (model 15 in Table 3) obtained from the combined data is as follows:

*True Hemoglobin value = 3.35 + 0.71 × Hemoglobin estimated POCT using capillary blood*

* POCT = invasive digital hemoglobinometer

The adjusted R2 value of the above equation (model 15 in Table 3) is 64.4%, which indicates that the above correction equation with hemoglobin values from POCT can explain 64.4% of the variability in the hemoglobin value obtained from AHA. The remaining 35.6% of the variability would be due to external factors other than the POCT.

We have also attempted to build prediction regression models (fixed ratio model) with a combination of POCT and other independent factors such as status of pregnancy, age, and gender to address the real-time scenario of the utilization of more than one type of POCT under national health programs (model 1 to 18 in Table 2 and Table 3).

The lowest R2 values are observed for the equation with the data only from HemoCue 201 among pregnant women without adjustment for any factors. We also observed the highest R2 values (>0.7) while using the devices Hemocue 301 (model 1–4) and TrueHb hemometer (9,10) in both adjusted and unadjusted regressions. The mean squared error of the hemoglobin estimated in POCTs ranged from 0.57 g/dL to 0.87 g/dL.

#### Predicted Hemoglobin vs. AHA Hemoglobin

We attempted to derive the mean difference (LOA) for the predicted hemoglobin values using the correction equation (model 15 in Table 3) and compared it against the hemoglobin levels estimated in AHA. The mean difference (LOA) is −0.01 (−1.8–1.8) g/dL (Figure 3).

### 3.2. Method 2—Rogan–Gladen Estimator

Using method (2), we also calculated the estimated true prevalence of anemia in India using NFHS-5 and CNNS data. The prevalence of a disease influences the sensitivity and specificity indicators of a diagnostic device. Therefore, the true prevalence obtained using the Rogan–Gladen estimator is influenced by the prevalence of the disease (Table 4).

## 4. Discussion

Accurate hemoglobin estimation is essential to assess the burden of anemia in the population. It is also critical to assess various causes of anemia and design geographically sensitive strategies. AHAs are the most commonly used technique for hemoglobin estimation in laboratories but with limited use in the field, peripheral sites, or large surveys, where POCTs are used. However, the invasive digital hemoglobinometers which are the widely used POCTs have shortfalls in accuracy compared with AHA or any other gold standard technique. Hence, a correction equation or a factor would help overcome this issue and mirror the values as the gold standard.

The cost of digital POCT devices ranges from USD 50 to 250. The per-test cost for consumables such as microcuvettes, lancets, and alcohol swabs will be less than USD 1. However, the cost of an AHA ranges from USD 600 to 6000. The per-test cost of hemoglobin estimation will be USD ~3–4. Compared to ANH, POCTs are less costly, portable, provide results immediately, and can be used by trained front-line workers. Considering these advantages, POCTs, if used effectively, can significantly change the burden of anemia in a country. Hence, the accuracy of hemoglobin values estimated in POCTs should be comparable with acceptable standards.

In this study, we attempted to emanate a correction equation as a first approach to predict the corrected hemoglobin values for POCTs using prediction statistics such as linear regression. We reviewed paired data from 1145 patients with hemoglobin estimated in (1) AHA using venous blood and (2) POCT with capillary blood. The mean difference in the hemoglobin values in AHA vs. POCT in the cumulative data was 0.2 g/dL and it ranged from −0.3 g/dL to 0.5 g/dL for individual POCTs. We arrived at the final simple model (model 15 in Table 3): “True Hemoglobin value = 3.35 + 0.71 × Hemoglobin estimated in invasive digital hemoglobinometer using the capillary blood”. This prediction equation has an R2 of 64.4%. The MSE between observed values and that of predicted values from the final linear regression model was 0.84 g/dL, which is well below the WHO accepted level of 1 g/dL for POCTs using capillary blood compared to AHA.

We considered the above model 15 (Table 3) as the final model because, first, the R2 values in the adjusted model with type of POCT alone (R2-0.659) and type of POCT along with other independent factors such as pregnancy, age, and gender (R2-0.667) were almost similar with the final model with only hemoglobin values (R2-0.644). Second, it may be cumbersome to adjust age, gender, and other unexplored parameters at this point in time for larger data, and a simpler approach can be explored in future research. Third, relatively higher R2 values were observed for two POCTs; however, they were not used in the DHS program (in India) so far. This article also opens windows of opportunity. When a validation study is inbuilt along with large population-based surveys, there is an option for adjusting for other independent factors that may affect the hemoglobin values. Additionally, in the Bland–Atman plot, the mean difference of the predicted hemoglobin using the correction equation vs. AHA is very small, −0.01 g/dL, compared to the mean difference of 0.2 g/dL in the original data (POCT vs. AHA).

A study from India compared the pooled capillary and venous blood hemoglobin levels in AHA and direct cyan-methemoglobin method, respectively. The mean difference (LOA) in capillary vs. venous blood hemoglobin estimated in AHA was −0.1 g/dL (−1.0 to 0.8 g/dL) and venous blood hemoglobin estimated in AHA vs. direct methemoglobin was −0.1 g/dL (−1.8 to 1.6 g/dL), respectively [20]. The capillary and venous blood hemoglobin levels were relatively closer while estimated in the AHA. This indicates that the capillary hemoglobin is closer to venous hemoglobin when estimated in the AHA. Hence, if the strict standard operating protocol was followed with POCTs, the error in hemoglobin estimation could be reduced further. We can also observe from Table 2 and Table 3 that Hemocue 201, which uses dried reagents and works on the principle of spectrophotometry, has relatively lower R2 and MSE values and would have impacted the model prediction. Hence, it is crucial that adequate training in POCT, controlling temperature and humidity, and using quality control timely may help estimate correct hemoglobin values, especially those which use reagents.

The second approach, the Rogan–Gladen estimator, is a relatively direct method to assess the true prevalence of anemia when the sensitivity and specificity of POCTs are available [21]. It also overcomes the issue of diagnostic misclassification or information bias by adjusting for imprecise accuracy estimates. This approach might also be helpful for the policy makers and program officers to derive corrected and scientifically credible anemia prevalence rapidly. We can observe from Table 4 that the estimated true prevalence is lower than the NFHS-5 prevalence and higher than the prevalence reported in the CNNS. In this study, we have used the sensitivity and specificity of the digital hemoglobinometers assessed in other studies and extrapolated them for NFHS prevalence. However, such validation exercises should be performed routinely in a subset sample of each demographic health survey. As the sensitivity and specificity of the POCT will have a linear relationship with the prevalence, the Rogan–Gladen estimator can be used for the whole of DHS data and also in situations where individual-level correction for hemoglobin is not feasible. However, we should be mindful of the fact that the Rogan–Gladen estimator can be misleading if the sensitivity and specificity are not from the study population surveyed for estimating the prevalence [22].

The limitation of this exercise could be that the trained laboratory technicians collected the hemoglobin values used in this study. The digital hemoglobinometers are designed for use by front-line workers such as auxiliary nurse midwives with minimal laboratory training. Hence, further validation of this model with front-line functionaries in the field settings will be required. We have included hemoglobin values of pregnant women, under-five children, 5–9-year-old school-going children, and adolescents (10–19 years old). Usage of this correction equation in other populations, such as adults and old age, may not be appropriate. We have adjusted the correction factor for age, gender, and pregnancy status. Other factors, such as genetic conditions of the individual, observer-related variations, and drop-to-drop variability in the capillary blood, are not adjusted and may have a role in estimating the correction equation.

However, the current correction factor formula explains 64% of the variability in predicting true hemoglobin level. Hence, we should accept that 36% of the variability is still not assessed. In a rapidly changing digital health technology environment, software upgradation or improvements in the digital hemoglobinometer technology is inevitable. Such changes may also affect the validity of the correction equation. This warrants further improvisation of the regression equation in parallel with the evolution of digital technology. The derived correction equation in the study is based on Indian studies. The final model can be validated in other countries to generalize the correction equation. We have not explored the hemoglobin estimated in other non-digital or non-invasive POCTs and their accuracy. The correction equation obtained in this research is intrinsic to the invasive digital hemoglobinometers, specifically to the three digital hemoglobinometers used in this study. Additionally, the validity of the correction factors for national, sub-national, and regional estimates is yet to be explored. Nevertheless, this is the first attempt to explore options to get a more accurate hemoglobin value or prevalence of anemia using digital hemoglobinometers as POCTs.

## 5. Conclusions

This study is an attempt to derive a correction factor for obtaining the true prevalence of anemia based on (1) regression models for hemoglobin levels comparable to the auto analyzer and (2) Rogan–Gladen estimation using sensitivity and specificity of the POCT. The first approach, though time-consuming, is better as it accounts for individual-level variations. The mean difference from the predicted hemoglobin using the correction equation was less than 0.01 g/dL. However, the second approach could be helpful when the validation study is part of the DHS programs. In addition, the type of POCT, procedure for estimation of hemoglobin, quality control of the POCT devices, and training level of the individuals can also affect the accuracy of correction factors in the estimation of true hemoglobin values.

## Figures and Tables

**Figure 1:**
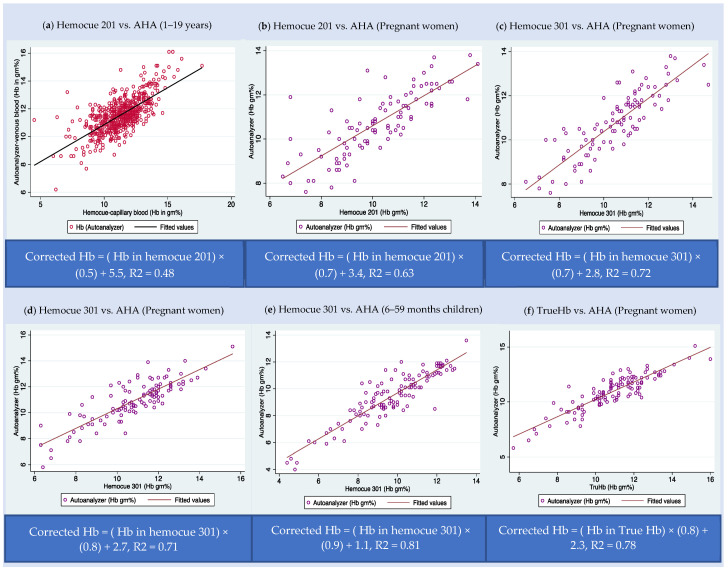
Scatter plot of hemoglobin estimated point-of-care tests (POCT) vs. automated hematology analyzers (AHA) based on the data subsets from India. (**a**) Hemocue 201 (POCT) vs. AHA among 1–19-year-old children—dataset D; (**b**) Hemocue 201 (POCT) vs. AHA among pregnant women—dataset A; (**c**) Hemocue 301 (POCT) vs. AHA among pregnant women– dataset A; (**d**) Hemocue 301 (POCT) vs. AHA among pregnant women—dataset B; (**e**) Hemocue 301 (POCT) vs. AHA among 6–59-month-old children—dataset C; and (**f**) TrueHb hemometer (POCT) vs. AHA among pregnant women—dataset B.

**Figure 2:**
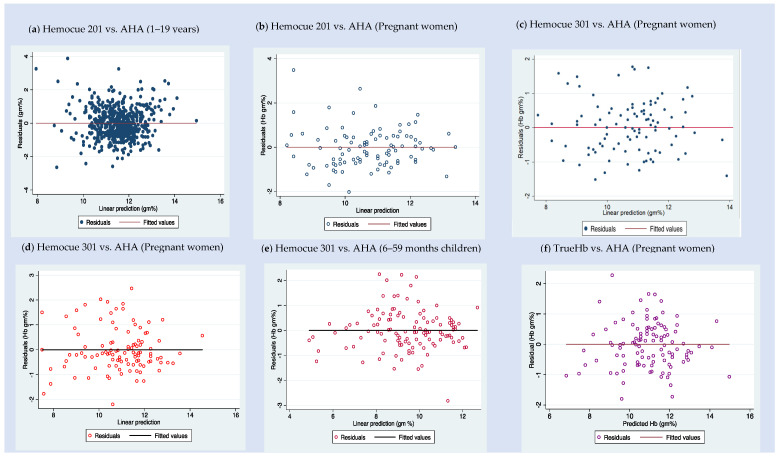
Plots of predicted vs. observed hemoglobin residual values for fixed ratio model based on the data subsets from India—residual plots of (**a**) Hemocue 201 (POCT) vs. AHA among 1–19-year-old children—dataset D; (**b**) Hemocue 201 (POCT) vs. AHA among pregnant women—dataset A; (**c**) Hemocue 301 (POCT) vs. AHA among pregnant women—dataset A; (**d**) Hemocue 301 (POCT) vs. AHA among pregnant women—dataset B; (**e**) Hemocue 301 (POCT) vs. AHA among 6–59- month-old children—dataset C; and (**f**) TrueHb hemometer (POCT) vs. AHA among pregnant women—dataset B.

**Figure 3:**
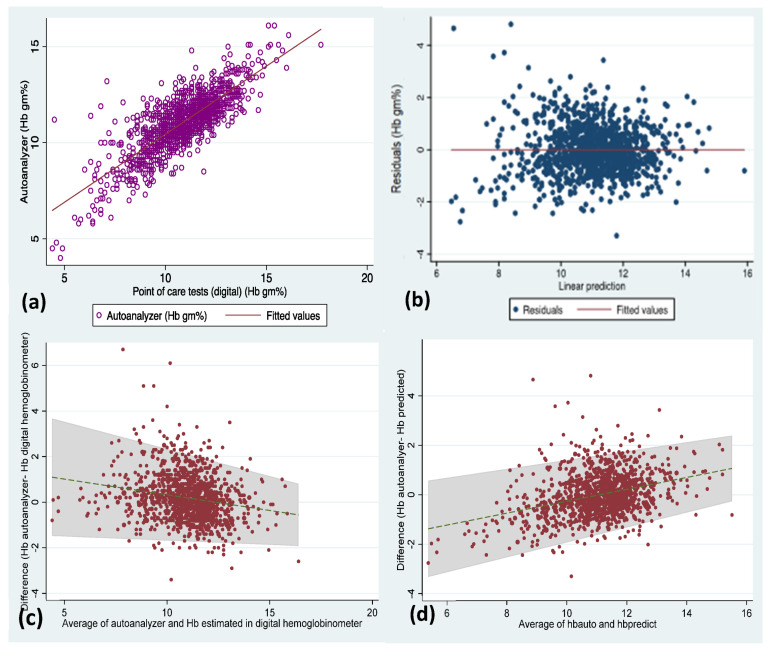
Scatter plot (**a**) for hemoglobin values estimated in all the POCTs against autoanalyzer, residual plot (**b**) of linear regression, Bland–Altman plot (**c**) depicting the mean difference in hemoglobin (Hb Autoanalyzer vs. Hb POCT) and limits of agreement for the whole data included in this study, and (**d**) Bland–Altman plot depicting the mean difference in hemoglobin (Hb Autoanalyzer vs. Hb predicted from correction equation).

**Table 1:** Overview of hemoglobin estimated in the automated hematology analyzers (reference or gold standard) and digital hemoglobinometers in the studies included in the calculation of correction factor.

Data	Author	Study Participants	n	Site of Study	Hemoglobin Estimated in AHA ^†^ Mean (SD) g/dL (A)	Point-of-Care Test		Mean Difference (95% CI) g/dL (A–B)
						Type	Mean (SD) of Test Hb g/dL (B)	
1	Ransi et al. (2020) [10]	1 to 19 years *	601	Community-based survey (CNNS)—dataset D	11.5 (1.2)	Hemocue 201	11.3 (1.6)	0.3 (0.2 to 0.3)
2	Ramaswamy G et al. (2020) [11]	Children (6 to 59 months) ^#^	120	Facility—dataset C	9.5 (1.8)	Hemocue 301	9.7 (1.9)	−0.3 (−0.4 to −0.1)
3	Yadav K et al. (2019) [12]	Pregnant women	102	Facility—dataset A	10.7 (1.4)	Hemocue 201	10.2 (1.7)	0.5 (0.3 to 0.7)
			102			Hemocue 301	10.5 (1.6)	0.2 (0.1 to 0.4)
4	Yadav K et al. (2020) [13]	Pregnant women	110	Facility—dataset B	10.9 (1.6)	Hemocue 301	10.8 (1.8)	0.1 (−0.1 to 0.3)
			110			TrueHb hemometer	10.9 (1.8)	0.04 (−0.1 to 0.2)

^†^ Reference method for hemoglobin—automated hematology analyzers (AHA). * Male: *n* = 307 (51.1%), Female: *n* = 294 (48.9%). ^#^ Male: *n* = 77 (64.2%), Female: *n* = 43 (35.8%).

**Table 4:** Comparison of the burden of anemia in NFHS-5 and CNNS data in India and estimated true prevalence using Rogan–Gladen estimator.

Age Groups	Prevalence in NFHS-5 (%)	Sensitivity (%)	Specificity (%)	Corrected Prevalence (Using Rogan–Gladen Estimator) (%)	Prevalence from CNNS (%)
6–59 months *	67	92.2	83.3	54.7	41
Adolescent girls 15–19 years ^#^	59	89.1	75.7	45.8	40
Adolescent boys 15–19 years ^#^	31	80.4	77.5	11.2	18
Women of reproductive age ^#^	57	92.8	75.0	41.3	NA
Pregnant women ^#^	52	93	76	38	NA
Lactating women	57	NA	NA	NA	NA

* Sensitivity and specificity for Hemocue 301 is available for this age group; ^#^ Sensitivity and specificity is available for Hemocue 201.

## Data Availability

Data are not available in the public domain.

## Supplementary Materials

The following supporting information can be downloaded at: https://www.mdpi.com/article/10.3390/diagnostics12123191/s1, Supplementary Figure S1. POCT—Hemocue 201+ with the reagent containing microcuvette. Supplementary Figure S2. POCT—Hemocue 301 with the non-reagent based microcuvette. Supplementary Figure S3. POCT—TrueHb hemometer with the reagent containing strip. Supplementary Figure S4: Automated hematology analyzer.

## References

[1] Safiri S., Kolahi A.A., Noori M., Nejadghaderi S.A., Karamzad N., Bragazzi N.L., Sullman M.J.M., Abdollahi M., Collins G.S., Kaufman J.S. (2021). Burden of Anemia and Its Underlying Causes in 204 Countries and Territories, 1990–2019: Results from the Global Burden of Disease Study 2019. J. Hematol. Oncol..

[2] Ministry of Health and Family Welfare, Government of India (2021). National Family Health Survey—5 (2019–2021). India Fact Sheet.

[3] World Health Organization Global Health Estimates: Leading Causes of DALYs. https://www.who.int/data/gho/data/themes/mortality-and-global-health-estimates/global-health-estimates-leading-causes-of-dalys.

[4] Plessow R., Arora N.K., Brunner B., Tzogiou C., Eichler K., Brügger U., Wieser S. (2015). Social Costs of Iron Deficiency Anemia in 6–59-Month-Old Children in India. PLoS ONE.

[5] Governnment of India, Ministry of Health and Family Welfare (2018). Anemia Mukt Bharat: Intensified National Iron Plus Initiative, Operational Guideliens for Programme Managers.

[6] Vitamin and Mineral Nutrition Information System, World Health Organization (2011). Haemoglobin Concentrations for the Diagnosis of Anaemia and Assessment of Severity.

[7] Srivastava T., Negandhi H., Neogi S.B., Sharma J., Saxena R. (2014). Methods for Hemoglobin Estimation: A Review of “What Works”. J. Hematol. Transfus..

[8] Sari M., de Pee S., Martini E., Herman S., Bloem M.W., Yip R. (2001). Estimating the Prevalence of Anaemia: A Comparison of Three Methods. Bull. World Health Organ..

[9] Neufeld L.M., Larson L.M., Kurpad A., Mburu S., Martorell R., Brown K.H. (2019). Hemoglobin Concentration and Anemia Diagnosis in Venous and Capillary Blood: Biological Basis and Policy Implications. Ann. N. Y. Acad. Sci..

[10] Abraham R.A., Agrawal P.K., Johnston R., Ramesh S., Porwal A., Sarna A., Acharya R., Khan N., Sachdev H.S., Kapil U. (2020). Comparison of Hemoglobin Concentrations Measured by HemoCue and a Hematology Analyzer in Indian Children and Adolescents 1–19 Years of Age. Int. J. Lab. Hematol..

[11] Ramaswamy G., Vohra K., Yadav K., Kaur R., Rai T., Jaiswal A., Kant S. (2020). Point-of-Care Testing Using Invasive and Non-Invasive Hemoglobinometers: Reliable and Valid Method for Estimation of Hemoglobin among Children 6–59 Months. J. Trop. Pediatr..

[12] Yadav K., Kant S., Ramaswamy G., Ahamed F., Jacob O.M., Vyas H., Kaur R., Malhotra S., Haldar P. (2020). Validation of Point of Care Hemoglobin Estimation Among Pregnant Women Using Digital Hemoglobinometers (HemoCue 301 and HemoCue 201+) as Compared with Auto-Analyzer. Indian J. Hematol. Blood Transfus..

[13] Yadav K., Kant S., Ramaswamy G., Ahamed F., Vohra K. (2020). Digital Hemoglobinometers as Point-of-Care Testing Devices for Hemoglobin Estimation: A Validation Study from India. Indian J. Community Med..

[14] Ministry of Health and Family Welfare, Government of India, International Institute for Population Sciences (2006). National Family Health Survey (NFHS-3).

[15] Indian Institute of Public Health; Government of India National Family Health Survey (NFHS 4). http://rchiips.org/nfhs/factsheet_NFHS-4.shtml.

[16] Ministry of Health and Family Welfare, Government of India CNNS Report—Nutrition India. http://nutritionindiainfo.in/rep_wp/cnns-report/.

[17] Hemocue AB Hemocue 301. https://www.hemocue.us/wp-content/uploads/2020/08/HB-301_Operating-Manual_US.pdf.

[18] Wrig Nanosystems TrueHb Hemometer Manual. https://5.imimg.com/data5/CP/OD/MY-4057306/true-hb-hemoglobin-meter.pdf.

[19] Hemocue AB Hemocue 201. https://www.hemocue.us/wp-content/uploads/2020/07/Manual_Glu_201.pdf.

[20] Dasi T., Palika R., Pullakhandham R., Augustine L.F., Boiroju N.K., Prasannanavar D.J., Pradhan A.S., Kurpad A.V., Sachdev H.S., Kulkarni B. (2021). Point-of-Care Hb Measurement in Pooled Capillary Blood by a Portable Autoanalyser: Comparison with Venous Blood Hb Measured by Reference Methods in Cross-Sectional and Longitudinal Studies. Br. J. Nutr..

[21] Rogan W.J., Gladen B. (1978). Estimating Prevalence from the Results of a Screening Test. Am. J. Epidemiol..

[22] Flor M., Weiß M., Selhorst T., Müller-Graf C., Greiner M. (2020). Comparison of Bayesian and Frequentist Methods for Prevalence Estimation under Misclassification. BMC Public Health.

