# Supplementary material for: Correction Equation for Hemoglobin Values Obtained Using Point of Care Tests—A Step towards Realistic Anemia Burden Estimates
Source: Diagnostics (Basel). 2022 Dec 16;12(12):3191. doi: 10.3390/diagnostics12123191 (PMC9777045; doi:10.3390/diagnostics12123191)
Supplement: Supplementary file 1 [file diagnostics-12-03191-s001.zip › diagnostics-2043315-supplementary.pdf]

Details of POCT devices:

HemoCue Hb 201+ is the second-generation digital POCT manufactured by Hemocue AB, Kuvettgatan, Ängelholm, Sweden. Hemoglobin measurement in this device is based on a modified glucose dehydrogenase method. The microcuvette has reagents containing chromogen compounds, and the erythrocytes are hemolyzed with saponin. The photometry absorbance is done at dual wavelengths (570 nm and 880 nm) to compensate for turbidity, and hemoglobin values are measured. The device's operating temperature range is 15–30 °C. This POCT takes 10–20 seconds to display the hemoglobin levels. (Supplementary Figure S1)

Hemocue AB, Kuvettgatan, Ängelholm, Sweden also develop HemoCue Hb 301. It uses non-reagent microcuvettes. The determination of hemoglobin values in Hemocue 301 is based on measuring the absorbance of whole blood at a Hb/HbO2 isosbestic point. It also uses double- wavelength measuring method, at 506 nm and 880 nm absorbance of whole blood at an oxyhemoglobin-deoxyhemoglobin isosbestic point. The device's operating temperature is 10–40 °C and the hemoglobin values are displayed within 10sec after loading the microcuvette with blood. In both Hemocue devices (201+ and 301) the micro cuvette also works like a pipette to draw blood (~10 micro ml) and a measuring cuvette to estimate hemoglobin. (Supplementary Figure S2)

True Hb is another digital POCT, it displays the hemoglobin results within 60 seconds, and the operating temperature range is wider from 5 to 55 °C. This device had a strip that contains reagent. The device's working principle is based on reflectance photometry following the conversion of hemoglobin to a complex with the help of a proprietary set of reagents present in the strip. (Supplementary Figure S3)

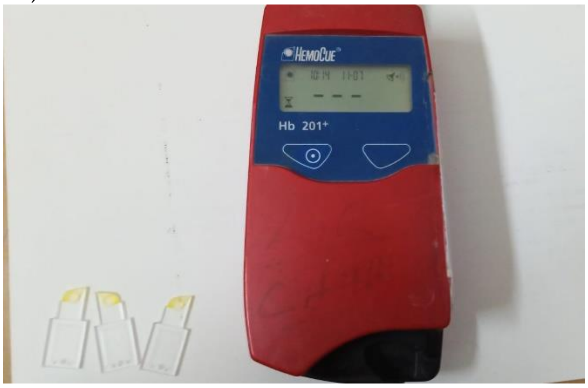

Supplementary Figure S1. POCT – Hemocue 201+ with the reagent containing microcuvette

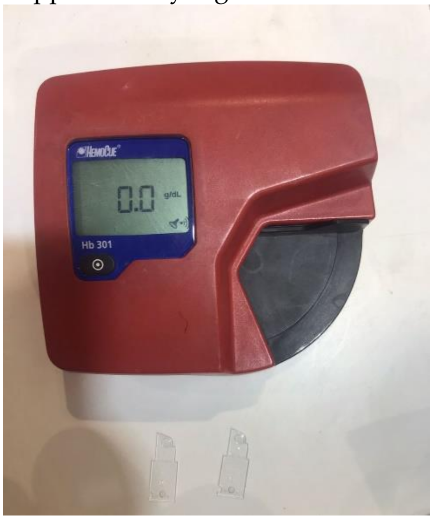

Supplementary Figure S2. POCT – Hemocue 301 with the non-reagent based microcuvette

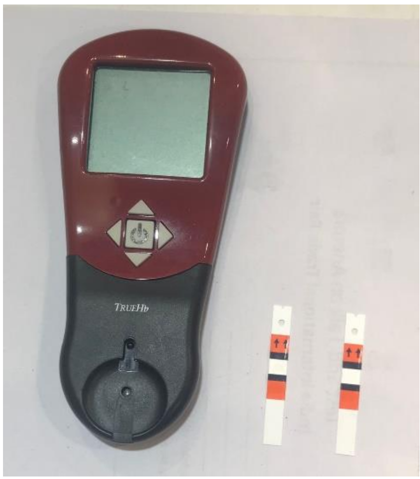

Supplementary Figure S3. POCT- TrueHb hemometer with the reagent containing strip
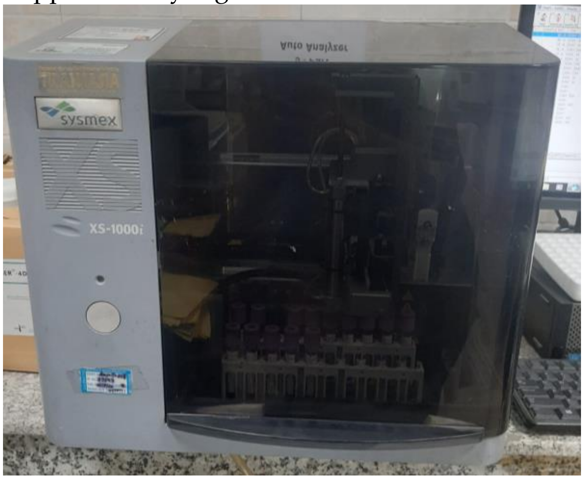

Supplementary Figure S4. Automated hematology analyzer
